# Development of a risk-stratification scoring system for predicting lymphovascular invasion in breast cancer

**DOI:** 10.1186/s12885-020-6578-0

**Published:** 2020-02-03

**Authors:** Ma-yi-di-li Ni-jia-ti, Di-li-a-re-mu Ai-hai-ti, Ai-si-ka-er-jiang Huo-jia, Pa-li-dan-mu Wu-mai-er, A-bu-du-ke-you-mu-jiang A-bu-li-zi, Yu Shi, Nu-er-a-mi-na Rou-zi, Wen-jing Su, Guo-zhao Dai, Mai-he-mi-ti-jiang Da-mo-la

**Affiliations:** Department of Radiology, The first people’s Hospital of Kashi area, No.120, Yingbin avenue, Kashi, Xinjiang Uygur Autonomous Region People’s Republic of China

**Keywords:** Breast cancer, Magnetic resonance imaging, Lymphovascular invasion, Risk stratification

## Abstract

**Background:**

Lymphovascular invasion (LVI) is a vital risk factor for prognosis across cancers. We aimed to develop a scoring system for stratifying LVI risk in patients with breast cancer.

**Methods:**

A total of 301 consecutive patients (mean age, 49.8 ± 11.0 years; range, 29–86 years) with breast cancer confirmed by pathological reports were retrospectively evaluated at the authors’ institution between June 2015 and October 2018. All patients underwent contrast-enhanced Magnetic Resonance Imaging (MRI) examinations before surgery. MRI findings and histopathologic characteristics of tumors were collected for analysis. Breast LVI was confirmed by postoperative pathology. We used a stepwise logistic regression to select variables and two cut-points were determined to create a three-tier risk-stratification scoring system. The patients were classified as having low, moderate and high probability of LVI. The area under the receiver operating characteristic curve (AUC) was used to evaluate the discrimination ability of the scoring system.

**Results:**

Tumor margins, lobulation sign, diffusion-weighted imaging appearance, MRI-reported axillary lymph node metastasis, time to signal intensity curve pattern, and HER-2 were selected as predictors for LVI in the point-based scoring system. Patients were considered at low risk if the score was < 3.5, moderate risk if the score was 3.5 to 6.0, and high risk if the score was ≥6.0. LVI risk was segmented from 0 to 100.0% and was positively associated with an increase in risk scores. The AUC of the scoring system was 0.824 (95% confidence interval [CI]: 0.776--0.872).

**Conclusion:**

This study shows that a simple and reliable score-based risk-stratification system can be practically used in stratifying the risk of LVI in breast cancer.

## Background

Identification of clinically predictive and prognostic factors is considered as an important issue in treatment evaluation of breast cancer. The first study on the prognostic significance of peritumoral lymphovascular invasion (LVI) in breast cancer was reported in 1964 [1], and not a few subsequent studies have reported the association between LVI and clinical outcomes of breast cancer. Accumulating evidence has showed that LVI has an unfavorable prognostic effect on breast cancer survival and recurrence across all molecular subtypes [2], and LVI is regarded as one of the crucial steps in breast cancer metastasis [3–5]. To date, breast cancer LVI is only available at pathological analysis. Preoperative knowledge of LVI can provide valuable information for determining the need for adjuvant chemotherapy or not.

Recently, a preliminary study used nomogram to predict the presence of LVI based on multiparametric magnetic resonance imaging (MRI) and pathological reports [6]. However, using a nomogram in clinical practice might be time-consuming and at risk of difficult interpretation. Klingen TA et al. reported that LVI was associated with HER2-positive and several features of aggressive breast cancer such as larger tumor size, higher histological grade, lymph node positive tumors and higher Ki67 expression [7]. Conventional MRI provides morphologic characteristics of the lesions with high spatial resolution, such as the margin morphology, or the internal architecture of the tumors [8]. Functional MRI such as diffusion-weighted imaging (DWI) in a fast time acquisition and without contrast medium gives information about cellularity of breast cancer [9]. Macchini M et al. found a correlation between dynamic contrast-enhanced (DCE) MR features with LVI [10]. Komatsu S et al. reported that a higher frequency of LVI was observed in the breast cancer lesions with the malignant pattern of time to signal intensity curve (TIC) [11]. This study aimed to develop a scoring system for stratifying the LVI risk of breast cancer based on pretreatment clinical, MRI and pathologic parameters.

## Methods

### Basic characteristics of patients

This retrospective study was approved by the Ethics Committee of the first people’s Hospital of Kashi area, and the patient’s informed consent was waived. We reviewed a total of 301 consecutive female patients (mean age, 49.8 ± 11.0 years; range, 29–86 years) with biopsy-proven solitary breast cancer admitted to the radiology department between June 2015 and October 2018. All patients underwent a 1.5 T MRI scan before surgery. Patients with complete clinicopathological characteristics. All patients had received no neoadjuvant therapy prior to operation. Patients with previously-diagnosed breast cancer or incomplete clinical records were excluded.

Clinicopathological features were collected including age, ER, PR, HER-2, Ki-67 index and histological subtypes. Positive ER or PR was recorded by immunohistochemistry when at least 1% of the tumor cell nuclei showed staining for ER or PR, respectively [12]. HER-2 was scored as 0, 1+, 2+ or 3+ in line with the American Society of Clinical Oncology/College of American Pathologists clinical practice guidelines, also adopted by the Italian Society of Pathological Anatomy and Diagnostic Cytology-Italian Division of the International Academy of Pathology (SIAPEC-IAP) [13]: 0, no staining observed or membrane staining that is incomplete or faint/barely perceptible in ≤10% of tumor cells; 1+, incomplete membrane staining that is faint/barely perceptible within > 10% of tumor cells; 2+, circumferential membrane staining that is incomplete and/or weak/moderate within > 10% of tumor cells, or complete and circumferential membrane staining that is intense within ≤10% of tumor cells; and 3+, circumferential membrane staining that is complete and intense within > 10% of tumor cells. The Ki-67 expression is defined as the percentage of positively stained tumor cells among the total number of malignant cells assessed [14]. The histological subtype of cancer was defined according to the World Health Organization classification.

### MR image acquisition

Preoperative breast MR imaging is a routine practice in our Institution. The MR imaging was performed in the prone position using a dedicated four-channel double-breast coil with a 1.5 T system (Siemens Avanto, Germany). Bilateral whole-breast MR imaging was performed using the following sequences and parameters: axial 3D gradient echo (GRE) Dixon T1-weighted sequence (TR/TE = 6.86 ms / 2.39 ms, section thickness = 2 mm, number of slices = 80, NEX = 1, matrix = 384 × 384, FOV = 360 × 360 mm, acquisition time = 2 min 19 s). Axial T2-weighted short tau inversion recovery (STIR) (TR/TI = 2550 ms / 170 ms, TE = 107 ms, section thickness = 5 mm, number of slices = 25, NEX = 4, matrix = 320 × 320, FOV = 350 × 350 mm, acquisition time = 2 min 33 s). Axial DWI was obtained with SE sequence (TR/TE = 6800 ms / 115 ms, section thickness = 5 mm, gradient directions = 3, matrix = 120 × 75, FOV = 350 × 350 mm, EPI factor = 107, bandwidth = 758, b values = 0, 800 s/mm^2^). Axial dynamic contrast-enhanced (DCE) MRI was performed using the volumetric interpolated breath-hold examination (VIBE) fat-suppression sequence (TR/TE = 3.0 ms / 1.42 ms, flip angle = 10°, matrix = 100 × 80, FOV = 150 mm × 120 mm × 70 mm), 55 dynamic phases, 7.3 s per phase, a total of 405 s. At the second phase of DCE scan, a bolus of 0.1 mmol kg^− 1^ of gadodiamide (Omniscan, GE Healthcare) was injected into the antecubital vein at a rate of 3 mL/s, followed by a 30 mL saline flush. After the DCE scan, axial and coronal T1-weighted fat-suppression contrast-enhancement sequences were obtained (TR/TE = 6.88 ms / 2.39 ms, section thickness = 2 mm, matrix = 384 × 384, FOV = 360 × 360 mm; TR/TE = 4.85 ms / 2.34 ms, section thickness = 3 mm, matrix = 320 × 320, FOV = 380 × 380 mm).

### MR image analyses

MR image analyses were performed on the picture archiving and communication system and Siemens post-processing workstation. Two radiologists with 10 and 20 years of experience with breast imaging, respectively, who were blinded to the pathological information, reviewed all MR images independently. If different assessments were assigned by the two radiologists, discrepancies were discussed to reach a consensus, and consensus was achieved between the two radiologists for all cases. MR image analyses included tumor location (upper-outer quadrant, upper-inner quadrant, lower-outer quadrant, lower-inner quadrant, or central position), tumor size (maximum diameter), margins (well- defined or ill- defined), lobulation sign (absence or presence), spiculation sign (absence or presence), MRI-reported axillary lymph node metastasis (ALNM) (absent, single or multiple), DWI appearance (slight/moderate hyperintensity or marked hyperintensity), time-intensity curve (TIC) patterns (type I, a straight or curved line; type II, a sharp bend after the initial upslope with plateau thereafter; type III, contrast washout was evident after an initial upslope). A 3 × 3 pixel standard-sized regions of interest (ROI) was selected within the areas of obvious intense enhancement in the tumor. TIC was generated. Metastatic axillary lymph nodes were nodes with major axis > 10 mm and hilus of lymph node disappeared.

### Reference standard

LVI was evaluated on routine hematoxylin–eosin-stained sections according to the method of Pinder et al. [15]. LVI was present if tumor cells were observed in the space of endothelial cells. Two pathologists with more than 10 years of experience reviewed all pathological images independently.

### Point-based scoring system development

We used a stepwise logistic regression to select variables. Variables in the stepwise analysis with *p* values < 0.05 were considered significant. For each significant variable a regression coefficient was obtained. Factors significantly associated with breast LVI in univariable and multivariable logistic regression analysis using stepwise method are listed in Table 1. Results of the univariable and multivariable logistic regression analysis were shown as odds ratio (OR) and corresponding 95% CI. Points for the prediction rule were assigned by doubling the value of the regression coefficients from the stepwise logistic regression and rounding to the nearest 0.5. In an effort to simplify eventual clinical application, a point-based scoring system was constructed using the method described by Sullivan et al. [16]. We then created cut points to classify patients as having low, moderate and high risk of LVI with optimum LVI rate for each category. To estimate the discriminating power of the scoring system, receiver operator characteristic (ROC) curves were created and the areas under the curves (AUCs) were calculated. The LVI rate in each category, according to pathology also determined, along with 95% confidence intervals (CIs). Specifically, we showed the use of point-based scoring system in the result section by taking two cases for example, one with LVI and another without LVI.

**Table 1:** Factors significantly associated with breast LVI in univariable and multivariable logistic regression analysis using stepwise method

Variable	Univariable regression		Multivariable regression	
	Odds Ratio (95%CI)	*p*-value	Odds Ratio (95%CI)	*p*-value
*Margins*				
Well-defined	reference		reference	
Ill-defined	4.99 (2.69–9.90)	< 0.001	3.46 (1.69–7.49)	0.001
*Lobulation sign*				
Absence	reference		reference	
Presence	4.00 (2.38–6.89)	< 0.001	3.66 (1.90–7.30)	< 0.001
*DWI appearance*				
Slight hyperintensity	reference		reference	
Marked hyperintensity	3.46 (2.02–6.15)	< 0.001	2.67 (1.42–5.17)	0.003
*MRI-reported ALNM*				
Absence	reference		reference	
Single	0.52 (0.26–1.04)	0.068	0.33 (0.14–0.75)	0.014
Multiple	0.26 (0.15–0.45)	< 0.001	0.26 (0.13–0.51)	< 0.001
*TIC type*				
I	reference		reference	
II	12.16 (2.50–219.37)	0.015	10.55 (1.87–201.14)	0.032
III	29.71 (5.55–553.11)	0.001	26.00 (4.07–520.41)	0.004
*HER2*				
-	reference		reference	
+	1.90 (0.61–7.24)	0.298	1.24 (0.31–5.89)	0.739
2+	2.76 (0.98–9.89)	0.078	2.48 (0.70–10.92)	0.195
3+	3.32 (1.11–12.41)	0.046	6.37 (1.64–30.54)	0.017

### Statistical analysis

R version 3.2.3 was used for statistical analysis. As for continuous variables, data were expressed as mean ± standard deviation (SD), while for categorial variables, data were expressed as counts and percentages (n, %). Continuous and categorial variables were compared by independent t tests, Mann-whitney U test, or Chi-square, if appropriate. The AUCs of logistic regression model and scoring system were compared with Delong test. A *p* <  0.05 were considered significant.

## Results

### Patient and tumor characteristics

Among the 301 lesions, 106 (35.2%) were invasive ductal carcinoma (IDC) (histological grade 3), 149 (49.5%) were IDC (histological grade 2), 46 (15.3%) were ductal carcinoma in situ (DCIS). In total, 104 (34.6%) patients (mean age, 49.6 ± 11.4) had LVI and 197 (65.4%) patients (mean age, 49.6 ± 11.4) without LVI. Comparison of clinicopathologic and radiological features between patients with and without LVI is shown in Table 2.

**Table 2:** Comparison of clinicopathologic and radiological features between patients with and without LVI

Parameters	No. of patients without LVI (*n* = 197)	No. of patients with LVI (*n* = 104)	*P* value*
*Age (years)*			
Mean	50.0 ± 10.8	49.6 ± 11.4	0.767
Median	49.0	48.0	–
< 50 (*n* = 167)	105 (62.9)	62 (37.1)	0.294
≥ 50 (*n* = 134)	92 (68.7)	42 (31.3)	
*Tumor location*			
Upper-outer quadrant (*n* = 14 6)	96 (65.8)	50 (34.2)	0.028
Lower-outer quadrant (*n* = 44 )	33 (75.0)	11 (25.0)	
Upper-inner quadrant (*n* = 74 )	51 (68.9)	23 (31.1)	
Lower-inner quadrant (*n* = 19 ) quadrant (*n* = 31)	11 (57.9)	8 (42.1)	
Central position (*n* = 18)	6 (33.3)	12 (66.7)	
*Tumor size (mm)*			
< 30 (*n* = 176)	124 (70.5)	52 (29.5)	0.030
≥ 30 (*n* = 125)	73 (58.4)	52 (41.6)	
*Margins*			
Well-defined (*n* = 95)	82 (86.3)	13 (13.7)	< 0.001
Ill-defined (*n* = 206)	115 (55.8)	91 (44.2)	
Lobulation sign Hyperechogenicity (*n* = 61)			
Absence (*n* = 135)	110 (81.5)	25 (18.5)	< 0.001
Presence (*n* = 166)	87 (52.4)	79 (47.6)	
*Spiculation sign*			
Absence (*n* = 122)	88 (72.1)	34 (27.9)	0.044
Presence (*n* = 179)	109 (60.9)	70 (39.1)	
*MRI-reported ALNM*			
Absent (*n* = 92)	43 (46.7)	49 (53.3)	< 0.001
Single (*n* = 51)	32 (62.7)	19 (37.3)	
Multiple (*n* = 158)	122 (77.2)	36 (22.8)	
*DWI appearance*			
Slight-moderate hyperintensity (*n* = 113)	92 (81.4)	21 (18.6)	< 0.001
Marked hyperintensity (*n* = 188)	105 (55.9)	83 (44.1)	
*TIC pattern*			
Type I (*n* = 25)	24 (96.0)	1 (4.0)	< 0.001
Type II (*n* = 229)	152 (66.4)	77 (33.6)	
Type III (*n* = 47)	21 (44.7)	26 (55.3)	
*ER (%)*			
< 50 (*n* = 116)	72 (62.1)	44 (37.9)	0.329
≥ 50 (*n* = 185)	125 (67.6)	60 (32.4)	
*PR (%)*			
< 50 (*n* = 191)	120 (62.8)	71 (37.2)	0.208
≥ 50 (*n* = 110)	77 (70.0)	33 (30.0)	
*HER2*			
- (*n* = 23)	19 (82.6)	4 (17.4)	0.132
+ (*n* = 63)	45 (71.4)	18 (28.6)	
++ (*n* = 149)	93 (63.3)	54 (36.7)	
+++ (*n* = 68)	40 (58.8)	28 (41.2)	
*Ki-67 (%)* index			
< 20 (*n* = 99)	64 (64.6)	35 (35.4)	0.838
≥ 20 (*n* = 202)	133 (65.8)	69 (34.2)	
*Histological type*			
IDC grade 3 (*n* = 106)	58 (54.7)	48 (45.3)	< 0.001
IDC grade 2 (n = 149)	98 (65.8)	51 (34.2)	
DCIS (*n* = 46)	41 (89.0)	5 (11.0)	

*Note*: unless otherwise indicated, data are numbers of nodules, and numbers in parentheses are percentages. *P value were calculated by using generalized estimating equation analysis. *DWI* diffusion-weighted imaging, *TIC* time-intensity curve, *ER* estrogen receptor, *PR* progesterone receptor, *HER2* human epidermal growth factor receptor, *IDC* invasive ductal carcinoma. *DCIS* ductal carcinoma in situ

### Development of point-based scoring system for predicting LVI

By multivariate logistic regression analysis, seven variables were significantly related to LVI and were assigned scores for the final prediction rule: margins (ill-defined [1.0 points], lobulation sign (presence [1.5 points]), DWI appearance (marked hyperintensity [1.0 points]), TIC pattern (type II [2.5 points], type III [3.0 points]), MRI-reported ALNM (single [− 1.0 points], multiple [− 1.5 points]), HER2 (+ [0 points], 2+ [1.0 points], 3+ [1.5 points]) (Table 3). If patients had < 3.5 points the probability of LVI was low with 5.3% (95% CI: 0.1–1.0%) having LVI. A score of 3.5–6.0 was moderate probability with 28.9% (95% CI: 21.7–36.2%) having LVI; a score of ≥6.0 was high probability with 76.7% (95% CI: 66.8–86.6%) having LVI.

**Table 3:** Points assigned to significant variables to determine patient score

Variables	Points
*Margins*	
Well-defined	0
ill-defined	1.0
*Lobulation sign*	
absence	0
presence	0.5
*DWI appearance*	
slight-moderate hyperintensity	0
marked hyperintensity	1.0
*TIC pattern*	
type I	0
type II	2.5
type III	3.0
*MRI-reported ALNM*	
absent	0
single	−1.0
multiple	−1.5
*HER2*	
-	0
+	0
2+	1
3+	1.5

### The performance of point-based scoring system for predicting LVI

**Fig. 1:**
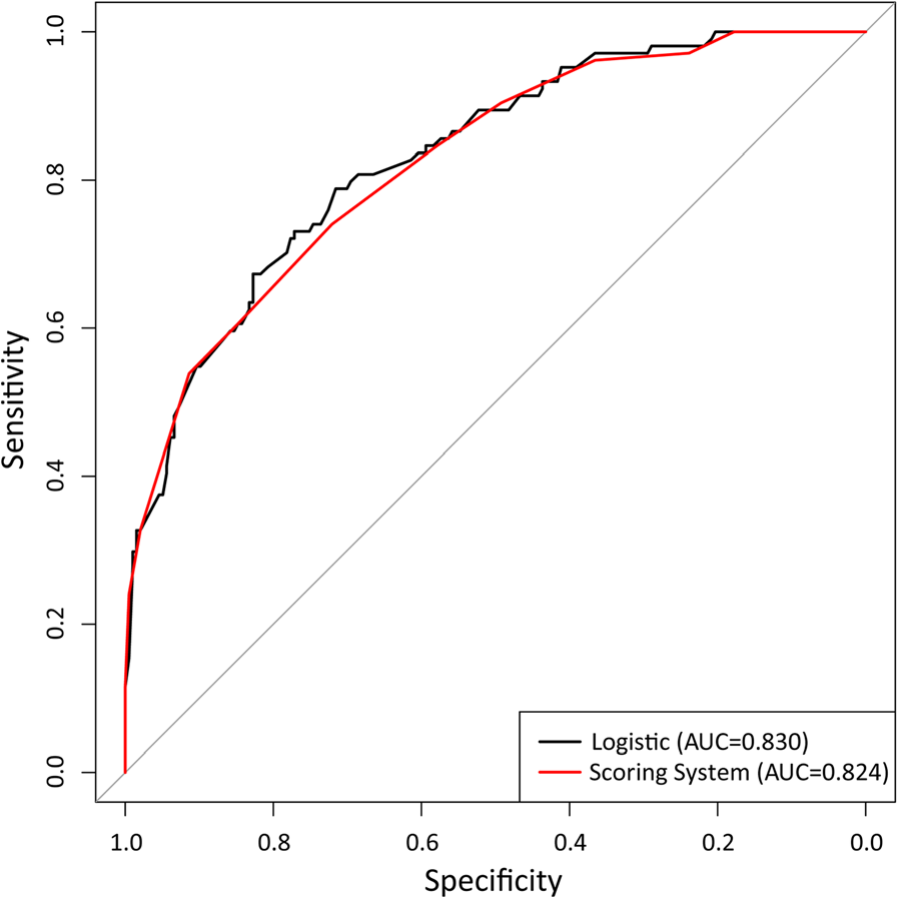
Receiver operator characteristic (ROC) curves comparison between the logistic regression model and scoring system. There were no significant AUC differences between the logistic regression model and the scoring system (*p* = 0.239)

The logistic regression model obtained an AUC of 0.830 (95%CI: 0.783–0.0.878). The proposed scoring system achieved an AUC of 0.824 (95%CI: 0.776–0.872). There were no significant AUC differences between the logistic regression model and the scoring system (*p* = 0.239) (Fig. 1). Table 4 presents the risk of LVI according to the point based scoring risk stratification system. LVI risk ranged from 0 to 100.0% and increased as the risk score increased and peaked at 100.0% in the scoring risk stratification model.

**Table 4:** Lymphovascular invasion risk according to the point-based scoring system

Risk score	LVI risk (%)	Total number	Number of LVI
−1.5	0%	1	0
−0.5	0%	2	0
0	0%	1	0
1	0%	1	0
1.5	0%	12	0
2	0%	4	0
2.5	0%	14	0
3	3.8%	15	3
3.5	19.4%	26	1
4	26.1%	31	6
4.5	28.2%	23	6
5	35.9%	39	11
5.5	35%	39	14
6	62.9%	20	7
6.5	75%	35	22
7	92.9%	12	9
7.5	100%	10	13
8	100%	2	10
Total	34.6%	301	104

### Examples of the risk-stratification scoring system in use

**Fig. 2:**
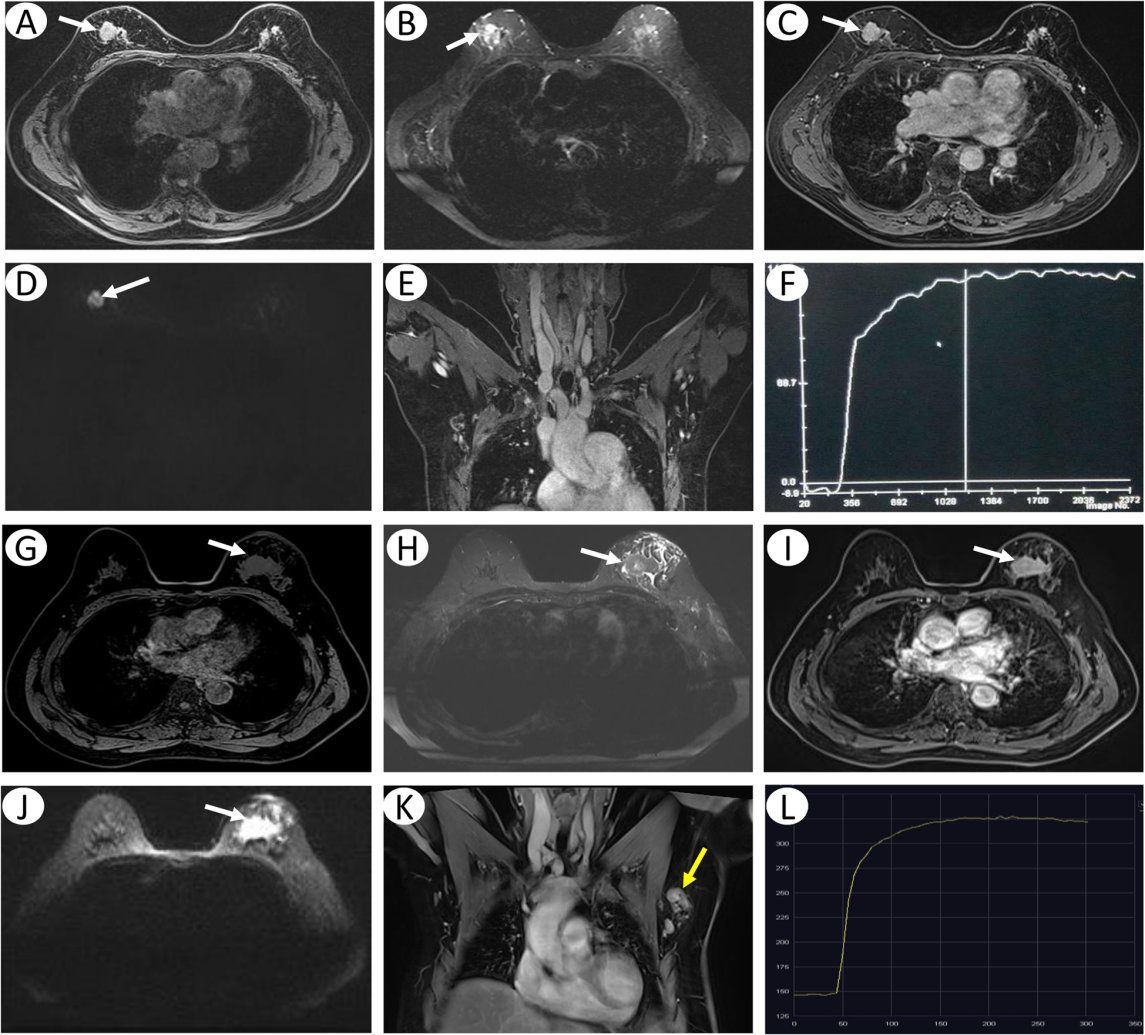
Examples of the scoring system in use. MRI examinations of patient 1 including. Axial T1-weighted fat-suppression (**a**), T2-weighted STIR (**b**), axial T1-weighted fat-suppression contrast-enhancement (**c**), DWI (**d**), TIC (**e**) and axillary imaging (**f**) showed a tumor (white arrows) with maximum diameter of 19 mm, location in the upper-outer quadrant, well-defined margins, absence of lobulation sign, obvious contrast-enhancement, marked hyperintensity on DWI images, type I TIC, and no ALNM. Biopsy results showed a IDC (grade 2) and positive HER-2 (+). The risk of LVI assessed by scoring system was 1. MRI examinations of patient 2 including axial T1-weighted fat-suppression (**g**), T2-weighted STIR (**h**), axial T1-weighted fat-suppression contrast-enhancement (**i**), DWI (**j**), TIC (**k**) and axillary imaging (**l**) showed a tumor (white arrows) with maximum diameter of 35 mm, location in the upper-inner quadrant, ill-defined margins, presence of lobulation sign, obvious contrast-enhancement, marked hyperintensity on DWI images, type II TIC, and multiple ALNM (yellow arrow). Biopsy results showed a IDC (grade 3) and positive HER-2 (3+). The risk score of LVI could be calculated to be 5 (Fig. 2n). Final pathology report showed this tumor had LVI

For example, patient 1 aged 59 years, who had a tumor in her right breast, location in the.

upper-outer quadrant, with maximum diameter of 19 mm, well-defined margins, absence of lobulation sign, marked hyperintensity on DWI images, type I TIC, and no ALNM (Fig. 2a-f). Biopsy results showed a IDC (grade 2) and positive HER-2 (+). The risk score of LVI assessed by our system was 1. Pathology report showed that the tumor was free of LVI. Patient 2 aged 57 years, who had a tumor in her left breast, location in the upper-inner quadrant, with maximum diameter of 35 mm, ill-defined margins, presence of lobulation sign, marked hyperintensity on DWI images, type II TIC, and multiple ALNM (Fig. 2g-l). Biopsy results showed a IDC (grade 3) and positive HER-2 (3+). The risk score of LVI could be calculated to be 5. Final pathology report showed that the tumor had LVI.

## Discussion

In this present study, we proposed a risk-stratification score system based on MRI-derived and histopathologic features to predict LVI in breast cancer. The system incorporated six predictors including tumor margins, lobulation sign, MRI-reported ALNM, DWI appearance, TIC pattern, and HER-2 status. Our results showed that the scoring system for predicting breast LVI had good discrimination (AUC = 0.831, 95%CI: 0.776--0.872). Within the system, patient’s risk for LVI was categorized as low (< 3.5 points), moderate (3.5–6.0 points) or high (≥6.0 points).

Risk stratification scoring system has been widely created to aid in clinical decision-making and potentially improve the patients management [17–22]. To our best of knowledge, this was the first study predicted LVI in patients with breast cancer using a comprehensive risk stratification scoring system. The scoring system was developed on the basis of a logistic regression model. According to the scoring system, the predicted risk of LVI ranged from 0 to 100%. For example, the risk of LVI is predicted to be 0% when a tumor had − 1.5 to 2.5 points and 100% when a tumor had 7.5 or 8 points. This system provides an easy tool for clinicians to evaluate the risk of LVI in breast cancer patients prior to surgery.

We found that MRI findings including tumor margins, lobulation sign, MRI-reported ALNM, DWI appearance, and TIC pattern were important risk factors for the prediction of breast LVI. We found tumor with LVI usually had morphological characteristics such as ill-defined margins, and lobulation sign. Tumors with irregular or spiculated or lobulated margins have a significantly higher microvessel density than tumors with smooth margins since the morphology is associated with a more aggressive behavior in breast cancer [23].

A review has shown that the presence of LVI correlates closely with locoregional lymph node involvement [24]. Although LVI was associated with MRI-reported ALNM, multiple ALNM was more frequent in tumors without LVI than those with LVI, which may due to the fact that LVI could occur before ALNM. Besides the conventional MRI, functional MRI such as DWI and DCE-MRI provides other important information about tumor diffusion and perfusion. Karan B et al. observed the median apparent diffusion coefficient (ADC) value was significantly associated with LVI (*P* = 0.008), which was in line with this study [25]. Malignant tumors have high cellularity but relatively less extracellular space, which has been shown to restrict water diffusion [26]. DCE-MRI has become a valuable tool to evaluate breast cancer morphologically and kinetically. Examination of the TICs allows physiological parameters related to tissue perfusion, microvascular vessel wall permeability and extravascular–extracellular volume fraction to be extracted, which may aid characterization of the underlying pathology. TIC type was the most important risk factor for LVI, the risk of LVI in tumors with TIC II and III was 10.6 and 26.0 times compared with tumors with TIC I, respectively. TIC III was more common in breast tumor with LVI. It may because TIC III is associated with peritumor inflammation. In addition, it suggests the presence of an increased vessel density and arterio-venous anastomoses with rapid outflow and thus fading of the contrast media [27]. HER-2 gene amplification or overexpression in breast cancer is a prognostic factor and predictive of a more aggressive clinical course for the patient. It is associated with high tumor-grade, hormone receptor-negative tumors, lymph node metastasis [28], increased risk of recurrence after surgery, poor response to treatment and shortened survival. This study found that the risk of LVI was significantly increased with the positive rate of HER-2 (*P*  <  0.001), which was in line with a previous study performed by Ugras et al. [29].

This study also has some limitations. Firstly, we evaluated DWI appearance instead of ADC values for LVI prediction because we didn’t measure ADC in routine practice. Secondly, this was a retrospective study. Thirdly, this study didn’t include multifocal or multicentric carcinoma. Finally, we didn’t test this scoring system in a validation cohort, which may limit the transfer of our system to other institutions.

## Conclusions

In summary, our MRI and HER2 based scoring system can be applied in a preoperative setting. The system can be used to stratify LVI risk in breast cancer patients, which is helpful for clinical decision-making regarding tumor staging, treatment planning, and prognosis assessment. Patients with LVI may require for adjuvant therapies because of the high risk of recurrence and metastasis.

## Data Availability

All data generated or analysed during this study are included in this published article.

## Abbreviations

ALVM: Axillary lymph node metastasis
AUC: Area under the curve
CI: Confidence interval
DCE: Dynamic contrast-enhanced
DWI: Diffusion-weighted imaging
LVI: Lymphovascular invasion
MRI: Magnetic Resonance Imaging
ROC: Receiver operating characteristic curve
TIC: Time to signal intensity curve
